# Recurrent variant c.1680C>A in *FAM20C* gene and genotype-phenotype correlation in a patient with Raine syndrome: a case report

**DOI:** 10.1186/s12887-021-02582-7

**Published:** 2021-03-06

**Authors:** Shruti Bajaj, Fazal Nabi, Jhanvi Shah, Harsh Sheth

**Affiliations:** 1 NH SRCC Children’s Hospital, 1-1A, Keshavrao Khadye Marg, Haji Ali, Haji Ali Government Colony, Mahalakshmi, Mumbai, Maharashtra 400034 India; 2 FRIGE’s Institute of Human Genetics, FRIGE House, Jodhpur Gam Road, Satellite, Ahmedabad, 380015 India

**Keywords:** Raine syndrome, FAM20C protein, Short life span, Extended life span, Case report

## Abstract

**Background:**

Bi-allelic mutations in *FAM20C* gene are known to cause a rare genetic disorder- Raine syndrome (RS). The FAM20C protein binds calcium and phosphorylates proteins involved in biomineralization of bones and teeth. RS is recognized as an osteosclerotic bone dysplasia. It is characterized by distinctive facial features, generalized osteosclerosis and respiratory insufficiency along with periosteal bone formation. RS is typically described as being an aggressive skeletal dysplasia with death in the neonatal period or early infancy. However, in the recent past an increasing number of individuals having an extended life span along with a highly heterogeneous phenotype has led to classifying RS into short and extended lifespan categories.

**Case presentation:**

We report a case of RS with antenatal fractures, facial dysmorphism and osteosclerosis without significant respiratory manifestations. The child has a relatively extended lifespan, whereby she died at 17-months of age. Clinical exome sequencing revealed a previously known, homozygous, nonsense variant c.1680C > A (p.Cys560Ter) in exon 10 of *FAM20C*. Whilst the variant was initially classified as a variant of uncertain significance (VUS), through the latest release of gnomAD and GTEx data, this was subsequently re-classified as likely pathogenic. Furthermore, segregation analysis showed both parents to be carriers. In contrast, a previously reported case with the same variant had polyhydramnios, complex facial abnormalities and bright echogenic brain parenchyma with oval shaped skull and anterior flattening at 26 weeks of gestation.

**Conclusion:**

The variant identified has been previously reported as a VUS. The present case provides further evidence towards the pathogenicity of the variant. A plausible genotype-phenotype correlation based on the location of the variant has been verified, wherein the position of a nonsense variant in the terminal exon of *FAM20C* gene, could have had a partial effect on the protein function, thereby resulting in a relatively milder phenotype and extended lifespan. Furthermore, the vast phenotypic variation on clinical comparison current case and a previously reported case, despite having the same genotype, could suggest an oligogenic effect and/ or environmental influence.

## Background

With a prevalence of less than 1:1,000,000, Raine syndrome (RS) (OMIM #259775) is an extremely rare genetic disorder [1]. A bi-allelic loss of function mutation in the *FAM20C* gene was first identified as the cause of RS in 2007, 15 years after its clinical description [2]. Clinical manifestations include generalized osteosclerosis, craniofacial dysplasia, thoracic hypoplasia and oro-dental anomalies. Developmental delay and neurological issues have also been observed in individuals with RS [2]. A marked increase in the ossification of the skull along with generalized increase in the density of all bones is observed on radiological evaluation. The increased ossification of the skull and facial bones underlies the characteristic facial features of RS, which include narrow prominent forehead, proptosis, depressed nasal bridge, and midface hypoplasia. Periosteal bone formation is also a characteristic feature of this disorder differentiating it from osteopetrosis and other known lethal and nonlethal osteosclerotic bone dysplasias. Severe midface hypoplasia and narrow thorax leading to choanal atresia and pulmonary hypoplasia respectively, contribute to respiratory distress and have been regarded as the causes for early death. RS has recently been classified into two subtypes based on the lifespan of the patient - lethal and non-lethal. Majority of the RS patients with an age ranging from 1 year to 61 years, with the so called non-lethal subtype showing highly variable phenotypic expression have been identified [1]. Of the 47 cases of RS reported worldwide; four are from India [1, 2]. RS is caused due to bi-allelic variants in the *FAM20C* gene having an autosomal recessive mode of inheritance. *FAM20C* gene, located on chromosome 7p22.3, spanning ~ 107 kb consists of 10 exons and encodes a protein of 584 amino acids in length. A critical kinase domain resides in its C-terminal, encompassing amino acids 354 to 565 [3]. To date, all RS patients have been identified with novel variants which have not been observed in other RS patients. We report here a unique case diagnosed with RS in which we identified a previously identified variant c.1680C > A (p.Cys560Ter) in exon 10 of *FAM20C*. With the latest population and gene expression databases, we re-classify the variant as likely pathogenic and perform genotype-phenotype analysis to assess the association of this variant in a relatively-extended lifespan phenotype.

## Case presentation

**Fig. 1:**
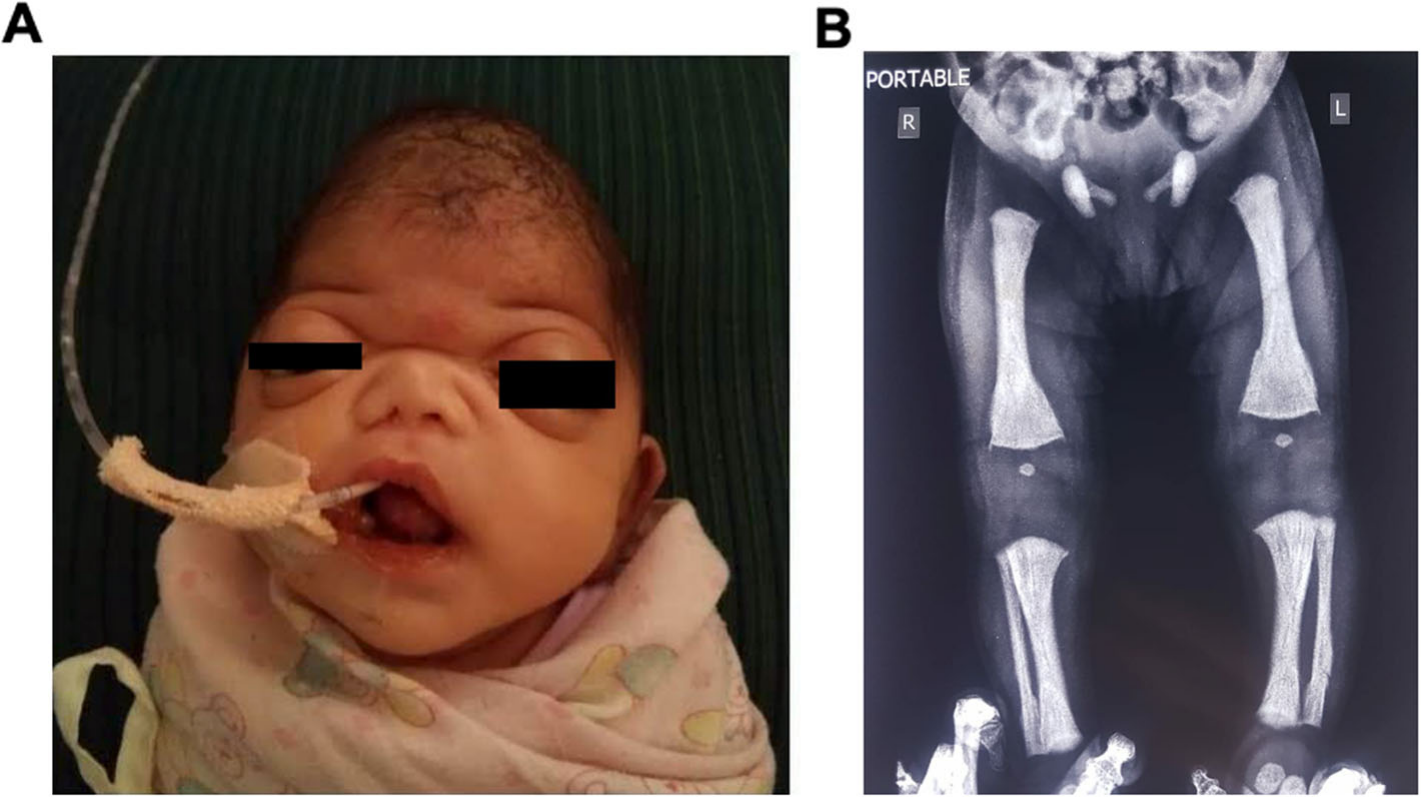
Clinical and radiographic profile of the child. **a** Facial photograph depicting gross dysmorphism. **b** Frontal view of the pelvic radiograph depicting generalized osteosclerosis, poor corticomedullary differentiation, microfractures in left femur and left fibula

A female child, second born to non-consanguineous parents, presented to the clinical genetics team on day-21 of life. Antenatal records showed an abnormal 32-week scan with clover-leaf skull, non-ossified nasal bone, midface hypoplasia, bulging eyelids and irregular contour of long bones. The child delivered at full-term, had Apgar score 9/10 and birth-weight of 2900 g. On presentation, the child had noisy breathing and feeding difficulties due to right-sided choanal atresia. Her vital parameters and oxygen-saturation were stable. Her weight, length and head-circumference were 2.75 kg (0 to − 2 Z-score), 46 cm (0 to − 2 Z-score) and 32.5 cm (− 1 to − 2 Z-score) on WHO growth-charts. Examination revealed bifrontal narrowing of the skull, bulging large anterior fontanelle, pronounced bilateral proptosis, severely depressed nasal-bridge, hypoplastic nose, anteverted nares, tented upper-lip, low-set ears, high-arched palate, hypertrophied gums, microretrognathia, dysplastic and loose natal teeth (Fig. 1a). The respiratory and cardiac examinations were normal. There was no visceromegaly. Review of previous investigations (day-2-of-life) revealed hypocalcemia (1.6 mmol/L), hypophosphatemia (1.49 mmol/L), low 25-(OH)_2_-vitamin-D (3.9 nmol/L) and normal complete-blood-count. Radiographs showed generalized osteosclerosis, poor cortico-medullary-differentiation and microfractures in left femur and fibula (Fig. 1b). Clinical differentials included RS and autosomal-recessive-osteopetrosis (ARO).

We performed clinical-exome-sequencing (CES) at an average depth of 100x on the germline-DNA extracted from peripheral blood. CES data analysis showed a previously identified, homozygous nonsense variant c.1680C > A (p.Cys560Ter) in exon 10 of *FAM20C* gene (ENST00000313766.5), which confirmed RS molecular diagnosis. In silico analysis revealed the variant to be absent in the 1000Genomes [4], ExAC and gnomAD databases [5], and predicted it to be damaging by DANN [5], FATHMM-MKL [6] and MutationTaster [7]. The variant had previously been detected in a non-related patient with RS and was classified as a variant of uncertain significance on the ClinVar database (SCV000608281.1) [8]. Based on this data, the variant was initially classified as a variant of uncertain significance according to the ACMG-AMP guidelines and ClinGen framework [9–11].

With the latest release of gnomAD genome and transcript specific annotation in the GTEx databases [12], we performed reanalysis of the variant. Analysis of the gnomAD database suggested the *FAM20C* gene to be intolerant to loss-of-function variants (pLEOUF = 0.48) and all reported loss of function variants in this gene in the population were heterozygous [13]. Transcript specific expression data in the GTEx database showed exon 10 of the *FAM20C* gene transcript being abundantly expressed in all tissues (median ptex score = 17.020) and coding for C-terminal-putative-kinase-domain of FAM20C protein, thereby having a critical role in protein function and conservation [14]. Hence, the variant c.1680C > A (p.Cys560Ter) was re-classified as likely pathogenic according to the ACMG-AMP guidelines and ClinGen framework [9–11] with following criteria- PVS1 (moderate), PM2 (moderate), PM3 (supporting), and PP4 (supporting).

**Fig. 2:**
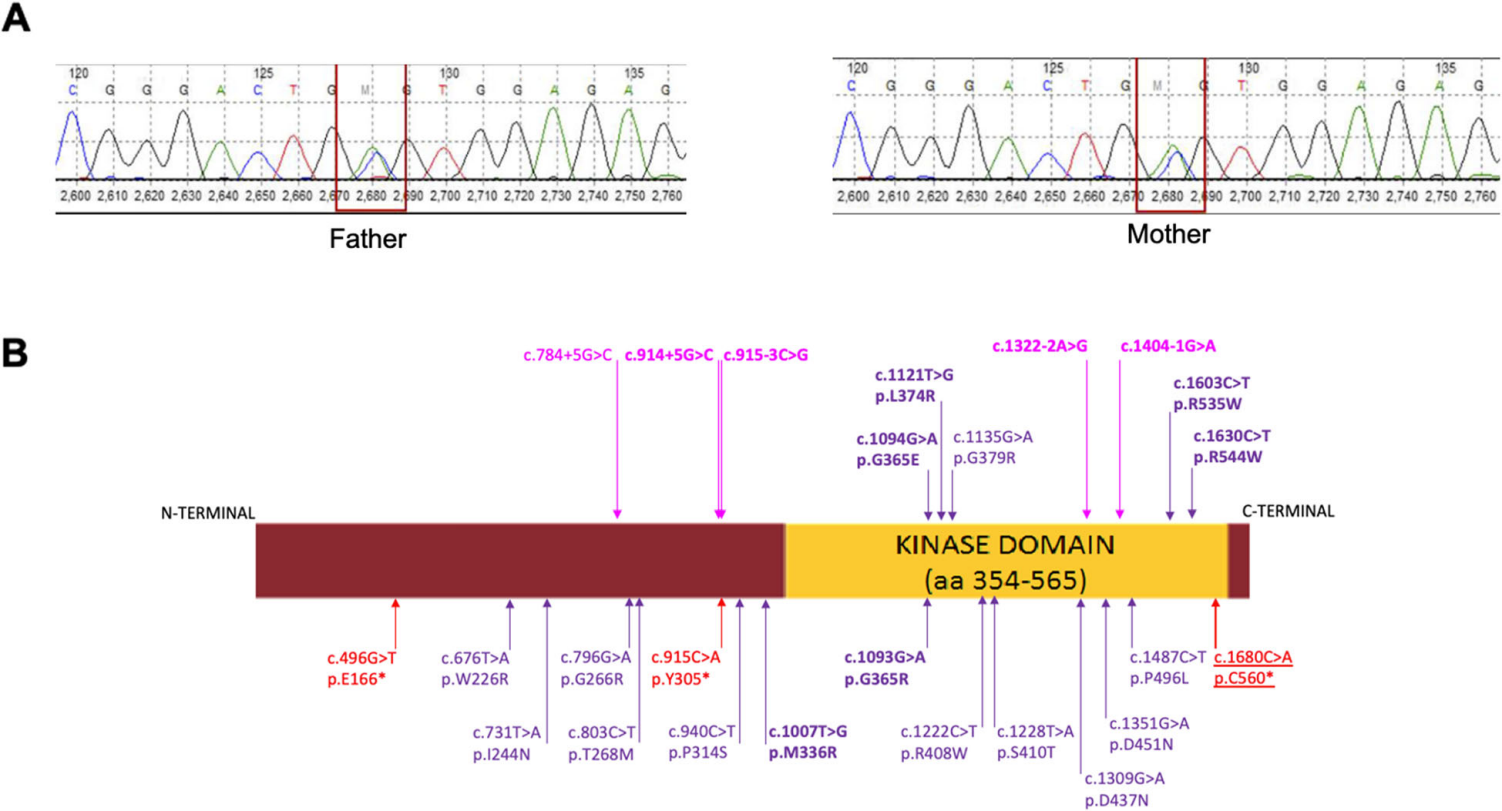
Molecular analysis of c.1680C > A in *FAM20C* gene. **a** Sanger sequencing chromatograms showing both parents as heterozygous carriers for the variant c.1680C > A in *FAM20C* gene. **b** Diagrammatic representation of the 25 known variants in the *FAM20C* gene which are detected in patients with Raine syndrome, along with protein domain information. Different types of variants are represented in different colours (missense- purple, nonsense- red and splice site- pink). Variants associated with neonatal or early infancy deaths are highlighted in bold and variant that is underlined is observed in the present case

Sanger sequencing for the aforementioned variant showed both parents to be heterozygous carriers (Fig. 2a). No transcriptome analysis in the proband could be carried out due to the absence of further peripheral blood samples, to assess the impact of the variant on mRNA decay or protein function. The family was offered genetic counseling about the prognosis, limited lifespan and 25% recurrence risk in all future pregnancies. They were satisfied with the closure and opted for minimalistic home-based care. They presented 20-months later, sharing the news of their daughter’s sudden demise at 17-months without any antecedent acute history (corroborated by a corresponding death certificate). Retrospective enquiry revealed that the child had feeding difficulties, poor growth, visual and auditory responses. She attained social smile by four-months, cooing by seven-months; other milestones were never achieved. Her natal teeth shed off by three-months; she remained edentulous thereafter. They denied seizures, respiratory distress or fractures in the child.

## Discussion and conclusions

RS is an extremely rare disorder caused due to homozygous or compound heterozygous mutations in *FAM20C* gene [1]. FAM20C protein helps in phosphorylation of intermediates that are important for normal bone and teeth bio-mineralization, thus explaining the skeletal phenotype of RS. Typical facial profile and osteosclerosis are important clinical clues to suspect RS early [2]. Fractures, osteosclerosis, choanal atresia, large anterior fontanelle, abnormal dentition, neurodevelopmental-delay, vision and hearing concerns are common to ARO and RS. ARO has some differentiating features as against RS: a) pancytopenia and hepatosplenomegaly, b) lacks typical facial profile, c) macrocephaly rather than microcephaly, d) lifespan in untreated ARO is around 10 years (lethal RS up to early infancy) and, e) cure available in form of hematopoietic stem-cell transplant (RS has none) [15]. Thus, need for a confirmed diagnosis, understanding the resultant implications on their child’s lifespan, treatment options’ availability and the prospect of precise prenatal diagnosis for the next pregnancy, made it relevant for the concerned family to seek a genetic diagnosis in this case.

Severely depressed nasal-bridge and midface hypoplasia contribute to choanal atresia, while narrow thorax leads to pulmonary hypoplasia; both ultimately causing pulmonary insufficiency and death, typically in the neonatal or early infancy, in RS [1, 2]. Of the four reported Indian cases of RS, one was an intrauterine-demise at 22-weeks, while the other succumbed to respiratory distress on day-38 [16].

Including the present case, we for the first time describe recurrence of the variant, c.1680C > A (p.Cys560Ter) in exon 10 of *FAM20C* gene in two unrelated patients from India. Here, we compare our case to the one carrying the same genotype and reported in the ClinVar database (SCV000608281.1) [8]. Our report based on the latest population allele frequency and gene transcript specific expression data reclassifies the previous identified variant as likely pathogenic. This previously identified variant describes a case of a fetus, product of a consanguineous marriage that presented with clinical indications of polyhydramnios. Fetal ultrasonography showed facial abnormality with absent nasal bone and protruding orbit suggestive of facial abnormality. Echogenic brain parenchyma with oval shaped skull and anterior flattening were also reported. Whether the pregnancy was terminated or was due to an intrauterine demise, is not reported. The couple had history of an abortion at 24 weeks’ gestation (details unknown) and also a male child who expired at one-month age (details unknown). Despite having the same genotype, the two cases show differences in their phenotypic expression, suggesting a possible role of additional genotypic effect and/ or environmental influence.

As of today, there are around 25 different variants reported that cause RS (Fig. 2b). These include missense, nonsense, and splice-site mutations that span the entire gene. Precise genotype-phenotype correlation has been difficult for RS, due to few cases and for the fact that most mutations are reported for the first time [2]. We have observed that majority of these mutations fall outside the critical kinase domain and belong to individuals showing an extended life-span except for the variant c.1007 T > G (p.M336R) reported by Hung et al. that exhibited neonatal death [17]. In contrast, most individuals with mutations residing within the kinase domain, specifically at its two terminal ends, have shown a shorter lifespan with death in neonatal period or early infancy (c.1135G > A (p.G379R) – status on lethality is unknown) (Fig. 2b). The variant observed in the present case, despite being a truncating variant and falling at the 3′ terminal end of the kinase domain, is associated with an extended lifespan in our case. The relatively prolonged survival up to 17-months in our case without respiratory distress could be postulated to be due to a relatively milder effect of the mutation because of its location at the distal end of the gene or due to an unknown oligogenic modifiers [18]. Genes with nonsense variants in the last exon have been observed to escape nonsense-mediated-mRNA-decay (NMD) [19, 20]. In the current case, presence of a nonsense variant in the final exon may have led to escape from NMD but since the exon codes for a critical protein domain, its functional effect is likely to be present. Hence, it is plausible that despite the presence of a nonsense variant, its location in the last exon of the *FAM20C* could have likely affected the protein function only partially, thus contributing to an extended lifespan up to 17-months. Alternatively, we can assume that another variant or environmental factors could have had an added preventive effect. Either or a combination of these hypotheses could have led to the relatively-extended lifespan in the present case.

## Data Availability

Not applicable.

## Abbreviations

RS: Raine syndrome
VUS: Variant of unknown significance
ARO: Autosomal recessive osteopetrosis
CES: Clinical-exome-sequencing
NMD: Nonsense-mediated-mRNA-decay
